# A Quantitative Method for the Specific Assessment of Caspase-6 Activity in Cell Culture

**DOI:** 10.1371/journal.pone.0027680

**Published:** 2011-11-29

**Authors:** Dagmar E. Ehrnhoefer, Niels H. Skotte, Jane Savill, Yen T. N. Nguyen, Safia Ladha, Li-Ping Cao, Edie Dullaghan, Michael R. Hayden

**Affiliations:** 1 Centre for Molecular Medicine and Therapeutics (CMMT), Department of Medical Genetics, CFRI, University of British Columbia, Vancouver, British Columbia, Canada; 2 Centre for Drug Research and Development (CDRD), Vancouver, British Columbia, Canada; 3 Institute of Cellular and Molecular Medicine (ICMM), Department of Medical Genetics, University of Copenhagen, Copenhagen, Denmark; McGill University, Canada

## Abstract

Aberrant activation of caspase-6 has recently emerged as a major contributor to the pathogeneses of neurodegenerative disorders such as Alzheimer's and Huntington disease. Commercially available assays to measure caspase-6 activity commonly use the VEID peptide as a substrate. However these methods are not well suited to specifically assess caspase-6 activity in the presence of other, confounding protease activities, as often encountered in cell and tissue samples. Here we report the development of a method that overcomes this limitation by using a protein substrate, lamin A, which is highly specific for caspase-6 cleavage at amino acid 230. Using a neo-epitope antibody against cleaved lamin A, we developed an electrochemiluminescence-based ELISA assay that is suitable to specifically detect and quantify caspase-6 activity in highly apoptotic cell extracts. The method is more sensitive than VEID-based assays and can be adapted to a high-content imaging platform for high-throughput screening. This method should be useful to screen for and characterize caspase-6 inhibitor compounds and other interventions to decrease intracellular caspase-6 activity for applications in neurodegenerative disorders.

## Introduction

Proteases of the caspase family are known as important mediators of apoptosis and have been commonly subdivided based on their roles in apoptosis or inflammation (apoptotic initiator, apoptotic executioner or inflammatory caspases). This definition however has become somewhat inaccurate as an increasing number of non-apoptotic roles for both initiator and executioner caspases have been identified that mediate cell differentiation, maturation and signaling events [1].

Caspases can further be distinguished based on their inherent differences in caspase substrate preference that are defined by the shape and electrostatic potential of the active site cleft [2]. Using positional scanning of peptide libraries, consensus recognition sequences have been proposed for each caspase and have led to the development of peptide substrates as well as inhibitors that typically consist of 4 amino acids (i.e. DEVD for caspase-3), followed by a fluorescent tag such as Afc (7-amino-4-trifluoro methylcoumarin) for a substrate or a ‘warhead’ such as fmk (fluoromethylketone) that covalently binds the enzyme for an inhibitor. These reagents are useful to investigate caspases that constitute the majority of caspase-like activity in a sample, as it may be assumed for active caspase-3 in highly apoptotic extracts [3]. However, with Km/kcat ratio differences of less than 10 fold for many widely used peptide substrates [4], these reagents are not particularly useful for investigating the activity of a caspase present at lower concentrations in cell culture and tissue samples. In particular in developmental or signalling processes that do not involve cell death, intracellular caspase activity is likely under tight control by endogenous caspase inhibitors or the proteasome [5], [6] and the resulting low levels of activity are difficult to detect with peptide substrates.

In biologic protease substrates, additional factors outside the 4 amino acid recognition site can influence the selectivity and efficiency of proteolytic cleavage. For caspases, it has been shown that the amino acid residue directly after the scissile bond (P1′) is an important determinant of cleavage, since charged or bulky residues are not well tolerated [7]. Furthermore, domains far away from the cleavage site can mediate the interaction between substrate and protease (exosites), and although such interactions have not yet been shown for proteases of the caspase family, the high variability of cleavage site motifs in natural caspase substrates argues in favour of the presence of exosites. Known substrates for caspase-6 show a particularly high variability in their recognition sequences [8], with cleavage sites other than I/D/E/L/T/V, E/D/Q, X, D found in substrates such as the presenilins (ENDD, [9]), huntingtin (IVLD, [10]), DNA Topoisomerase I (PEDD, [11]), AP-2 alpha (DRHD, [12]), Periplakin (TVAD, [13]), FAK (VSWD, [14]) and TGEV (VVPD, [15]).

Caspase-6 has garnered much attention recently since it has been shown that it is involved in the developmental pruning of axons [16], [17], and it has been suggested that similar pathways might erroneously be activated in neurodegenerative disorders such as Alzheimer's (AD) and Huntington disease (HD) [16], [18]. The presence of activated caspase-6 and cleavage of caspase-6 substrates is indeed a hallmark of AD, HD and cerebral ischemia, and has been shown in a number of different animal models and patient brain tissue [18], [19], [20], [21], [22]. To assess caspase-6 activity in cell and tissue samples, peptide substrates or inhibitors need to be titrated accurately to yield meaningful results, since the peptide substrate commonly used to assess caspase-6 activity, VEID, can be cleaved by other caspases as well as the proteasome when used at too high concentrations [23], [24]. In addition, even low concentrations of a VEID substrate can lead to inaccurate results if the relative amount of other proteolytic activity in the sample is significantly higher than that of caspase-6 due to small differences in kcat/Km [4]. A recently developed method using the biotinylated caspase inhibitor zVAD is significantly more specific, but to achieve this specificity, immunoprecipitation and subsequent detection of the active caspase by Western blotting is required [23]. For a more quantitative way of assessing caspase-6-specific activity, we have developed a novel method based on the cleavage of lamin A [24]. Here we show that this method is sensitive, specific and allows for the detection of caspase-6 activity in complex samples. The assay quantitatively measures caspase-6 activity and can be useful for investigating caspase-6 biology as well as the screening and intracellular efficacy assessment of caspase-6 inhibitors.

## Results

In order to develop an activity assay that is more specific for caspase-6 than available methods relying on the cleavage of the VEID peptide, we decided to investigate the cleavage of known caspase-6 substrates. To be useful for the measurement of caspase-6 activity, a substrate would ideally be as specific as possible. In particular it should not be cleaved by other proteases activated during apoptosis and be cut by caspase-6 in detectable quantities. The protein substrate should be easy to purify and thus available in larger amounts, and existing neo-epitope antibodies allowing for the specific detection of the cleavage fragment would be an advantage. The nuclear lamin proteins were among the first caspase-6 substrates characterized [25], and knockdown studies strongly suggest that their cleavage during apoptosis is highly dependent on the presence of caspase-6 [24]. Furthermore, purified lamin A as well as a variety of neo-epitope antibodies against the cleavage fragments are readily available, and high stability of the cleavage fragments without further degradation during the apoptotic process has been described [26] , which should facilitate their detection.

The VEID peptide sequence commonly used in caspase-6 substrates and inhibitors is derived from the lamin cleavage site, but since protein context is known to change the kinetics and specificity of proteolytic events, we decided to compare the cleavage of the VEID peptide to that of the full-length lamin A protein.

### The lamin A protein is a more specific substrate than its VEID peptide

**Figure 1:**
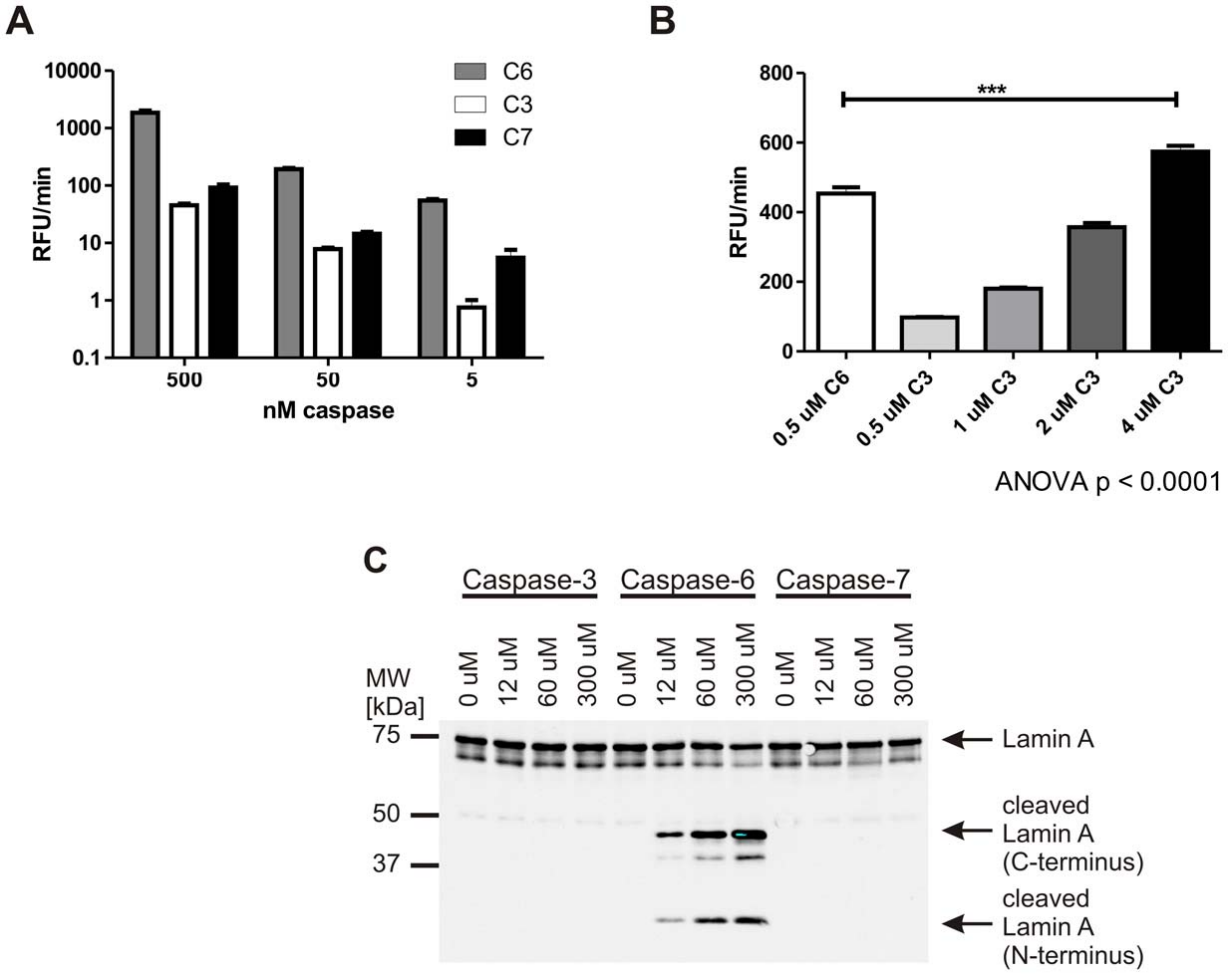
The lamin A protein is a more specific substrate than its VEID peptide. **A:** 100 µM VEID-Afc was incubated with different amounts of caspase -3, -6 or -7 for 1 h at 37°C. Fluorescence generated by cleavage was monitored over time, and the initial, linear portion of the curve was used to calculate the reaction velocity. VEID is preferentially cleaved by caspase-6, but also cross-reacts with caspases -3 and -7 at higher concentrations. Error bars are the SEM of N≥3 of 3 independent experiments. **B:** 5 µM VEID-Afc was incubated with 0.5 µM caspase-6 or different amounts of caspase-3 for 1 h at 37°C. The reaction velocity was calculated as in A. Even at this low concentration of VEID-Afc, the peptide substrate can be cleaved by caspase-3, and an 8 fold higher molar concentration of caspase-3 than caspase-6 results in a higher signal for VEID cleavage by caspase-3 than caspase-6. Error bars are the SEM of N = 3 independent experiments, statistical significance was assessed by 1-way ANOVA and post-hoc Dunnett comparisons: *** p<0.0001. **C:** Pure lamin A protein was incubated with different amounts of caspase -3, -6 or -7 for 30 min at 37°C. Samples were separated by SDS-PAGE and both fragments of cleaved lamin A was detected by Western blotting with antibodies #2031 (full-length lamin A and N-terminal fragment) and #2032 (C-terminal fragment). No cleavage was observed with caspases -3 or -7, while caspase-6 generated lamin A fragments in a dose-dependent manner. A representative image of 3 independent experiments is shown.

We therefore decided to investigate whether lamin A is a specific substrate for caspase-6. In particular, we were interested in the ability of other executioner caspases to cleave lamin A, since these are likely to show high activity in apoptotic extracts where accurate quantification of caspase-6 activity will be of interest. We first subjected the Ac-VEID-AFC substrate to digestion by caspases -3, -6 and -7, respectively. The amount of active enzyme used in each reaction was normalized to the concentration of active sites in the sample as determined by titration against the irreversible inhibitor zVAD-fmk (Fig. S1). As expected, the VEID peptide substrate, although it is best processed by caspase-6, still shows significant cleavage by caspase-3 and -7 (Fig. 1A) at high substrate concentrations (100 µM). Lower substrate concentrations can be used to achieve greater specificity, however, the difference in kcat/Km for VEID is less than 3 fold between caspase -3 and -6 [4]. This results in a small concentration window that can be exploited to achieve selectivity for caspase-6, if the same amounts of active caspase-3 and -6 are present. However, this window will be lost if the sample contains a higher concentration of active caspase-3 than caspase-6. As shown in Figure 1B, an 8 fold molar excess of caspase-3 over caspase-6 is enough to lead to a significantly higher signal from the non-specific caspase-3, making VEID-based assays problematic for the use in apoptotic samples or other cell and tissue lysates with high levels of active caspase-3. Lamin A, on the other hand, only showed proteolytic processing when incubated with recombinant active caspase-6, not with the corresponding amounts of caspases -3 and -7 at concentrations up to 300 µM (Fig. 1C). We therefore focussed on lamin A as a substrate for the development of a specific caspase-6 activity assay.

**Figure 2:**
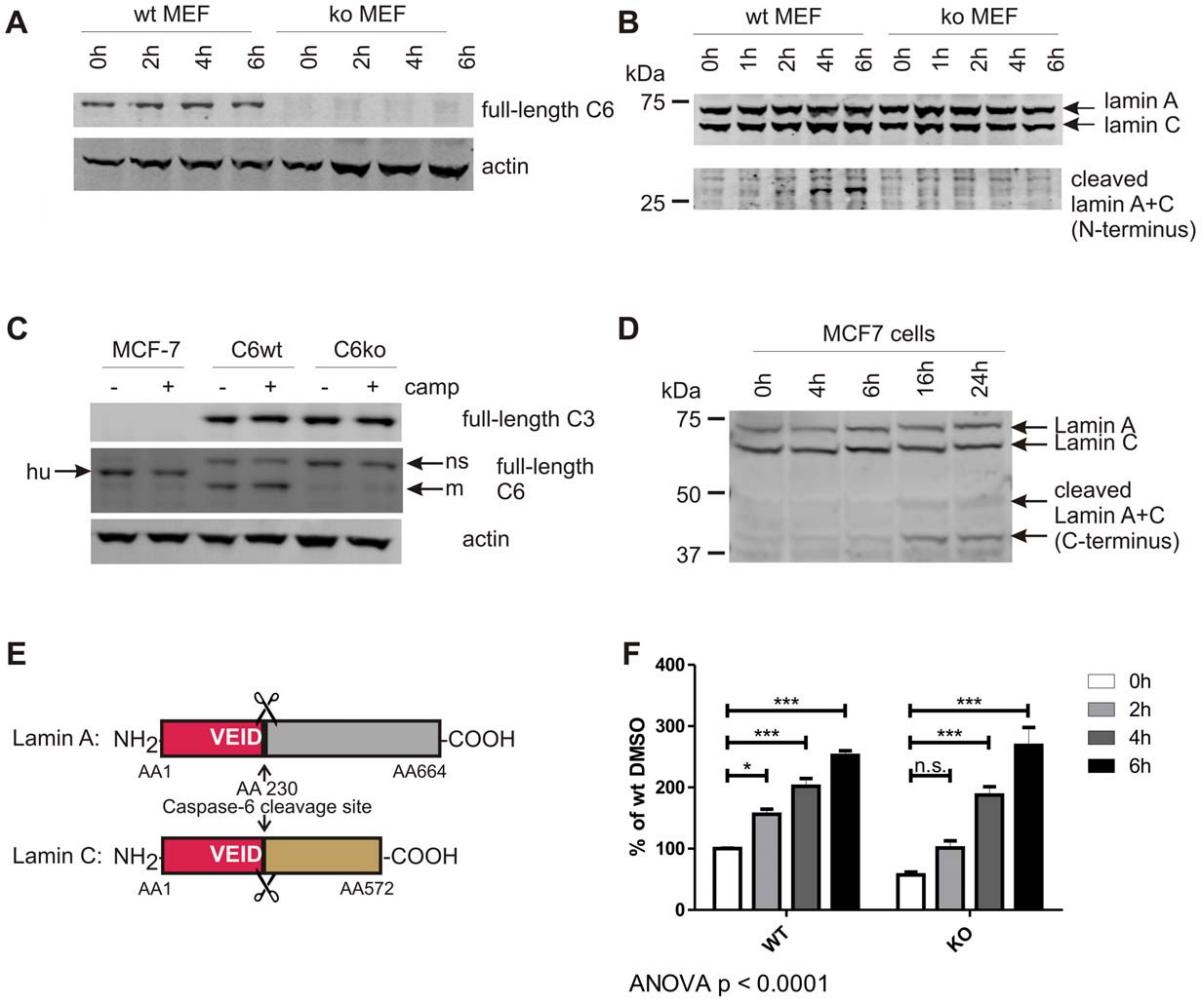
VEID, but not lamin A+C, is cleaved in the absence of caspase-6. **A:** Caspase-6 protein (full-length, 32 kDa) is detected in MEFs generated from C6wt, but not C6ko mice. **B:** Endogenous lamin A protein (70 kDa) is cleaved in wt, but not C6ko MEFs after staurosporine stress for 4 h or longer. The antibody cross-reacts with full-length lamin C (60 kDa), and the cleaved band at 28 kDa has the same size for both lamin A+C (lower panel). **C:** MCF-7 cells express caspase-6, but not caspase-3 protein, whereas both C6wt and C6ko MEFs contain both caspases. hu: human, m: mouse, ns: non-specific band. **D:** MCF-7 cells were stressed with 5 µM camptothecin for different amounts of time and the cleavage of endogenous lamin A and C proteins was monitored by Western blotting with antibodies antibodies #2031 (full-length lamin A+C) and #2032 (C-terminal fragments). **E:** Schematic representation of lamin A and C and the caspase-6 cleavage site at AA 230. The N-terminal fragments generated by caspase-6 cleavage (red) have the same size (28 kDa) for both lamin A+C. **F:** C6wt or C6ko MEFs were stressed with 50 nM staurosporine for different amounts of time, lysates were generated and analyzed for cleavage of VEID-Afc. C6wt cells show a significant increase in fluorescence at each timepoint, the fluorescence signal obtained from C6ko lysates only reach a statistically significant difference from baseline after 4 h. C6ko MEFs stressed with staurosporine for 4 h or more show the same levels of VEID proteolysis as wt cells. Error bars are the SEM of N = 3 of 4 independent experiments. Statistical significance was assessed by 2-way ANOVA and post-hoc Bonferroni comparisons: *** p<0.0001, ** p<0.001, * p<0.01.

To confirm the selectivity of lamin cleavage by caspase-6, we made use of mouse embryonic fibroblast (MEF) cells generated from wild-type and caspase-6−/− (C6wt and C6ko) mice [27]. These cells only differ in their expression of caspase-6 (Fig. 2A), making them an ideal system to study the specificity of caspase-6 substrates. Apoptosis was induced by the addition of 50 nM staurosporine to the culture medium, and the activation of caspase-6 was monitored over time by assessing the cleavage of endogenous lamin A via Western blotting (Fig. 2B) or with the Ac-VEID-AFC peptide substrate (Fig. 2F). The antibody used to detect full length lamin A at 70 kDa cross-reacts with the closely related lamin C protein (60 kDa, [28]), and the N-terminal fragments of both cleaved lamin A and C are detected at the same size of 28 kDa (Fig. 2B+E). Furthermore, using a cell line that does not express caspase-3 but expresses caspase-6 (Fig. 2C), we found that the lamin proteins are still processed after the induction of apoptosis with camptothecin (Fig. 2D), confirming the requirement of caspase-6 but not caspase-3 for this process.

An increase in VEID cleavage over time was observed for both C6wt and C6ko cell lines (Fig. 2F). For C6wt cells, significant increases over baseline were observed at all time points, whereas the C6ko cells show lower VEID processing initially with a non-significant increase in the first 2 h of treatment. However, at later timepoints C6ko cells exhibit similar levels of VEID cleavage as C6wt with a highly significant increase over non-treated controls (Fig. 2F), suggesting that other proteases make up for a large proportion of the VEID cleavage activity in apoptotic extracts. These results indicate that the lamin protein substrates are much more specific for cleavage by caspase-6 than the VEID peptide.

### Development of an electrochemiluminescence-based ELISA method for the quantitative assessment of cleaved lamin A

**Figure 3:**
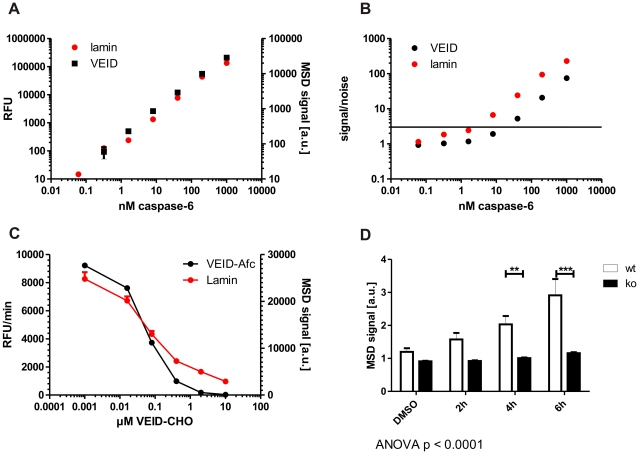
Increased sensitivity and highly improved specificity in a lamin-based caspase-6 activity assay. **A+B:** Quantities of down to 10 nM active caspase-6 can be detected with the novel lamin A-based caspase-6 assay while maintaining a signal-noise ratio of >3. **C:** The peptide inhibitor VEID-CHO shows a similar IC_50_ value in both the VEID- and the lamin A- based caspase-6 assays. **D:** The lamin A-based caspase-6 assay is highly specific, with no signal generated from C6ko cells even after 6 h of staurosporine stress. Error bars are the SEM of N≥3 of 3 independent experiments. Statistical significance was assessed by 2-way ANOVA and post-hoc Bonferroni comparisons: *** p<0.0001, ** p<0.001.

In order to accurately quantitate the amount of cleaved lamin A generated by caspase-6, we turned to an ELISA-based assay format using the Mesoscale® platform, which allows for a fast and sensitive detection with minimal background using electrochemiluminescence [29]. To determine whether cleavage of lamin A is more sensitive than cleavage of the VEID peptide substrate in detecting low levels of active caspase-6, we incubated different concentrations of the enzyme in parallel either with Ac-VEID-AFC or purified lamin A protein and determined the amount of cleavage after 30 min by measuring the fluorescence in the sample (for Ac-VEID-AFC) or by subjecting the sample to an ELISA using the lamin A neo-epitope antibody (for lamin A cleavage). Comparison of the results showed that both assays perform at least equally well in detecting active caspase-6 concentrations down to 10 nM, with the lamin cleavage assay showing a linear concentration-response relationship down to the lowest caspase concentrations tested (Fig. 3A). Signal-to-noise ratios were at or above 3 for the ELISA assay for as low as 10 nM caspase-6, whereas the signal-to-noise dropped below 3 for the VEID-based assay at this concentration (Fig. 3B). Using a peptide inhibitor for caspase-6, VEID-CHO, both assays furthermore arrived at a similar IC_50_ value (lamin-based ELISA: 64±20 nM, VEID-based assay: 56±6 nM), indicating that the ELISA method is suitable to assess the inhibition of caspase-6 by small molecules or peptides. Overall, our method shows a slight increase in sensitivity over commonly used VEID cleavage methods and is able to reliably detect caspase-6 concentrations down to 10 nM.

Next, we wanted to assess whether the lamin cleavage assay shows higher specificity than the VEID-based system for caspase-6 over other proteases that are activated after induction of apoptosis. To this end, we tested lysates derived from staurosporine-stressed C6wt and C6ko MEFs with our newly developed ELISA and found a linear increase in signal over time in wt samples, whereas the signal from C6ko samples remained stable at background levels that were similar to the background seen in untreated C6wt (Fig. 3D). This indicates that the amount of intracellularly cleaved, endogenous lamin A protein is a highly specific readout for the quantification of caspase-6 activated during staurosporine-induced apoptosis.

Kinetic measurements of either Ac-VEID-AFC or lamin A cleavage by fluorescence or our newly developed ELISA allowed comparison of the kinetic parameters of caspase-6 for the two substrates. We find that the full-length lamin A protein has a more than 1000fold lower Km than its cleavage site peptide VEID (Table 1). Although the kcat value is also decreased, the kcat/Km is still more than 10fold higher for lamin A (Table 1). This indicates that the binding between lamin A and caspase-6 is tighter, which might be mediated by domains outside the cleavage site. Such binding sites could also be responsible for the observed specificity of caspase-6 for the full-length protein substrate. The Km and kcat values for VEID are furthermore in good agreement with previously reported data [4].

**Table 1:** Km and kcat values for caspase-6 substrates.

	Km	kcat	kcat/Km
VEID	30.9±2.2 µM	4.3±0.12 sec^−1^	139 200 M^−1^ sec^−1^
lamin A	14.11±2.592 nM	0.057±0.0025 sec^−1^	4 055 043 M^−1^ sec^−1^

### Quantitation of caspase-6 activity in neuronal lysates

Caspase-6 is postulated to be involved in neuronal degeneration and apoptosis [16], [17], [18], [20], [22], [30], and detection of its activity in neuronal cultures is therefore of paramount interest. Previous studies have frequently used TUNEL assays as a measure of apoptosis, looked at VEIDase activity or the presence of the active caspase-6 fragment by Western blotting or immunostaining and assessed the presence of cleaved caspase-6 substrates [8], [18], [20], [21], [22]. The most specific methods to assess the effect of drug treatments on caspase-6 activity developed so far use antibodies against the active caspase-6 fragment, levels of which would not necessarily change upon inhibitor treatment, or immunoprecipitation of active caspases with biotinylated zVAD-fmk, which depends on efficient precipitation and Western blotting for quantification [23].

**Figure 4:**
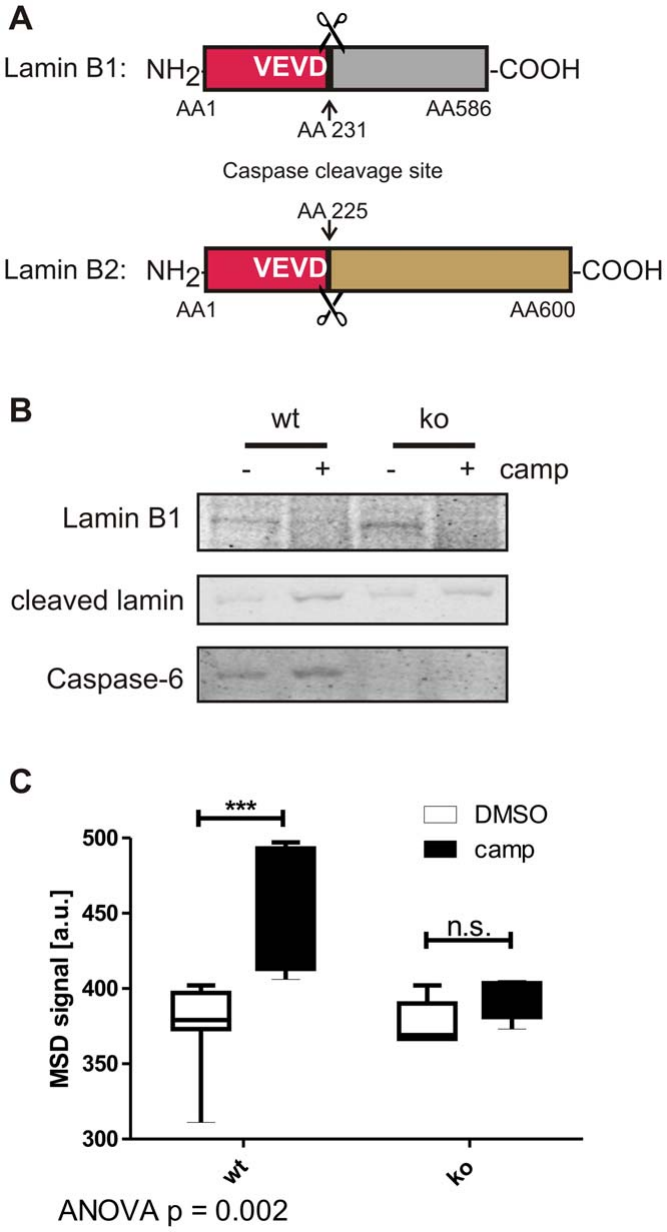
Cleavage of lamin B1 in primary neuronal cultures is not caspase-6 specific, but spiking with lamin A provides a specific readout. **A:** Lamins B1 and B2 differ from lamins A and C in their caspase cleavage site (VEVD versus VEID, respectively). **B:** Lamin B1 is cleaved after camptothecin stress in primary cortical neuronal cultures derived from C6wt and C6ko mice. **C:** Spiking with lamin A protein results in an increased ELISA signal in lysates from camptothecin-stressed C6wt, but not C6ko neurons. Boxes represent the 25^th^–75^th^ percentile, whiskers represent the minimum and maximum. Data from 5 (C6ko) and 7 (C6wt) separate cultures are shown. Statistical significance was assessed by 2-way ANOVA and post-hoc Bonferroni comparisons: *** p<0.0001.

Our newly developed ELISA method, however, could not be directly applied to neuronal cultures, since lamin A is not expressed in embryonic mouse brain [31]. Neurons in early stages of development up until postnatal day 5 express lamins of the B1 and B2 subtypes, which differ from the lamin A and C sequence at the caspase cleavage site (VEVD in B-type lamins and VEID in lamins A and C (Fig. 4A) [24], [31]). Furthermore, the cleavage of lamins B1 and B2 at this site is not specific for caspase-6, as has been shown in a caspase-6-deficient cell line [24]. In agreement with these findings, we observed cleavage of lamin B1 in primary cortical neurons derived from both C6wt and C6ko mice at embryonic day 16.5 (Fig. 4B), while lamins A and C were not detected (data not shown). To overcome this obstacle, we decided to spike C6wt and C6ko neuronal lysates with purified lamin A protein, and after incubation at 37°C to allow for its cleavage by endogenous caspase-6 activity, we subjected the samples to our ELISA assay. We detect a significant increase in cleavage of lamin A protein when the incubation was performed in the presence of extracts derived from camptothecin-stressed C6wt neurons, whereas no increase in cleaved lamin A signal was observed in the presence of camptothecin-stressed C6ko neuronal extracts (Fig. 4C). The ELISA method is therefore also suitable to detect caspase-6 activity in samples that do not contain endogenous lamin A or C protein.

### Detection of caspase-6 activity in the absence of endogenous lamin cleavage

**Figure 5:**
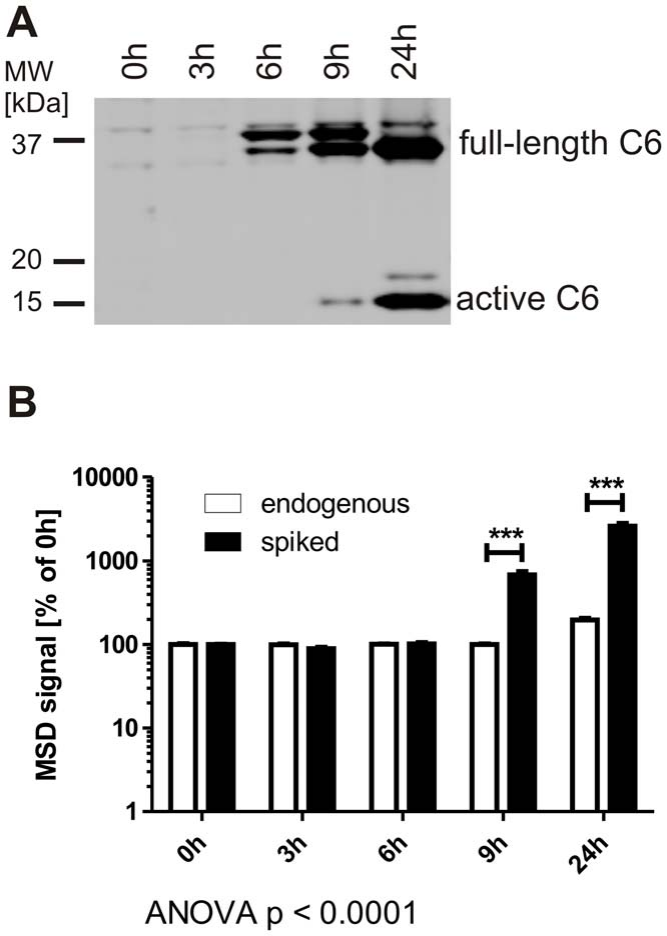
Spiking with lamin A allows for the detection of caspase-6 activity before the cleavage of endogenous lamins. **A:** COS7 cells were transiently transfected with recombinant human caspase-6 and cultured for the indicated amounts of time. The active caspase-6 fragment can be observed by Western blotting 9 h after transfection and is increasingly abundant after 24 h. **B:** COS7 cells were transfected as in **A**. The cleavage of endogenous lamin A protein can be observed with the ELISA method after 24 h, whereas spiking of lysates with lamin A protein leads to a robust detection of caspase-6 activity 9 h after transfection. Statistical significance was assessed by 2-way ANOVA and post-hoc Bonferroni comparisons: *** p<0.0001.

Caspase-6 activity can be localized to the nucleus, which is commonly associated with cell death and the cleavage of nuclear substrates such as lamin A, whereas a cytoplasmic localization of active caspase-6 as it is the case in neurodegeneration does not result in immediate apoptosis [5], [21], [32]. We therefore decided to test whether spiking of cell extracts with pure lamin A protein can detect active caspase-6 at early timepoints, before its translocation to the nucleus and cleavage of endogenous lamin A. To this end we transiently transfected COS7 cells with full-length human caspase-6, a system in which the enzyme auto-activates in the cytosol before translocating to the nucleus [32]. After transfection, the active form of caspase-6 becomes detectable by Western blotting at the 9 h timepoint (Fig. 5A). After 24 h of incubation, low levels of endogenous lamin A cleavage were observed with the ELISA method, indicating that at this time point active caspase-6 is present in the nucleus (Fig. 5B). However, when cell lysates were supplemented with purified lamin A protein and endogenous, active caspase-6 was allowed to cleave the spiked protein, activity could already be detected after 9 h and increased dramatically at later time points (Fig. 5B), in agreement with the Western blot data (Fig. 5A). This indicates that the spiking method can detect caspase-6 activity before endogenous lamin is cleaved and is thus suitable to detect non-nuclear caspase-6 activity.

### High-throughput immunofluorescence measurements of lamin A cleavage

**Figure 6:**
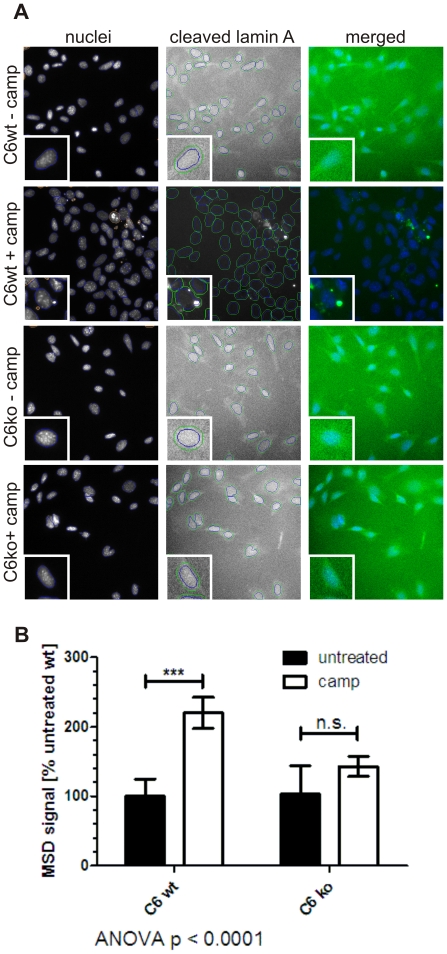
Caspase-6 activity can be quantified by immunofluorescent staining for cleaved lamin A. **A:** C6wt and C6ko MEFs were stressed with camptothecin, stained with an antibody against cleaved lamin A and analyzed on an automated imaging platform. Nuclei were counterstained with DAPI, and identified by the software (blue circles). Debris (red circles) was not analyzed. The signal intensity in the cleaved lamin A channel was quantified in a ring around the nucleus (green circles). **B:** Quantitation of the perinuclear staining from cleaved lamin A is graphed. Untreated C6wt MEFs were normalized to 100% and the fold change in stressed C6wt, stressed and non-stressed C6ko MEFs are compared. Error bars are the SEM of N≥3 of 3 independent experiments. Statistical significance was assessed by 2-way ANOVA and post-hoc Bonferroni comparisons: *** p<0.0001.

Although our Mesoscale ELISA method shows significant advantages over commonly used, VEID-based assay systems to measure caspase-6 activity, it still requires manual cell lysis and protein quantification steps that are not easily amenable to high-throughput screening campaigns. We therefore turned to a high-content imaging platform that allows automated liquid handling as well as immunofluorescence imaging and quantification. Using a primary antibody specific for caspase-6 cleaved lamin A, we found increased perinuclear staining in C6wt cells after induction of cell death by camptothecin (Fig. 6A). The observed blebbing of the nuclear membrane is consistent with the breakdown of the nuclear lamina during apoptosis. No staining for cleaved lamin protein was observed in non-stressed C6wt, non-stressed C6ko or camptothecin-stressed C6ko cells (Fig. 6A). Using nuclear DAPI staining as a reference point, we quantified the perinuclear immunofluorescence in a ring around the nucleus (Fig. 6A, insets), and found a 2fold increase in staining intensity in camptothecin-stressed versus non-stressed wt MEFs (Fig. 6B). Only background signal was observed in C6ko MEFs even after camptothecin treatment, indicating that the detection of cleaved lamin A by immunofluorescence is also specific to caspase-6 (Fig. 6B). This method is ideally suited for the high-throughput intracellular testing of modulators of caspase-6 activity in in the presence of confounding proteolytic activity, i.e. that of caspase-3.

## Discussion

The study of the role of caspases during apoptosis, but also during non-apoptotic developmental and signalling processes, is hampered by the lack of specific assays to measure the activity of single members of the caspase family. Neo-epitope antibodies against the active forms of caspases are available and can be used in immunohistochemical and Western blotting applications. However, the presence of active caspase fragments does not necessarily correlate with proteolytic activity, since the proteases could be bound to either endogenous or exogenous inhibitors, which is especially problematic in high-throughput screening campaigns aiming to identify caspase inhibitors. Another method using immunoprecipitation with biotinylated zVAD, on the other hand, is specific to active caspases, but quantitation relies on the efficacy of immunoprecipitation and Western blotting [23].

To be able to accurately quantify the activity of caspase-6 in cell culture, we therefore developed novel activity assays based on the cleavage of the caspase-6 specific substrate protein lamin A [24]. Here, we confirm the specificity of lamin A cleavage by caspase-6 using C6wt and C6ko MEFs that were stressed with staurosporine to undergo apoptosis. Although the antibodies we used in this study cross-react with cleaved lamin C, specificity for caspase-6 cleavage is maintained, since both proteins arise from the same gene by alternative splicing, contain the same caspase-6 cleavage site and only differ in their C-termini with lamin A being 10 kDa longer (Fig. 2D) [28], [33]. The cleaved fragment was detected in C6wt, but not in C6ko MEFs, and we developed an electrochemiluminescence-based ELISA method to accurately quantify cleaved lamin using the Mesoscale platform. The new method has an improved detection limit and signal-noise ratio over commercially available caspase-6 activity assays using the VEID peptide substrate. Furthermore, our assay is specific to caspase-6 and thus superior for the detection of caspase-6 activity in complex samples with a variety of different proteolytic activities such as cell lysates. Through spiking with purified lamin A protein, the assay can be applied to samples lacking endogenous lamin A and C such as embryonic primary neurons. Furthermore, the method is amenable to samples where caspase-6 activity does not lead to the cleavage of the endogenous nuclear lamins and is therefore not necessarily associated with apoptosis.

The specificity is due to the use of a protein instead of a peptide substrate, and even though the VEID sequence is derived from the caspase-6 cleavage site in lamin A (aa227–230), we show that the Kcat/Km value for lamin A is more than 10fold higher than for VEID, indicating that lamin A is a better substrate for caspase-6. A similar effect has been shown for proteases involved in blood coagulation [34], [35], where substrates are bound outside the active site of the protease (exosite). There has been speculation about the presence of exosites in caspases [36], but their existence has not been proven conclusively yet. The example of exploiting protein substrate specificity for the development of an activity assay could be used for the development of more specific assays for other members of the caspase family, such as p23 for caspase-7 [3].

Caspase-6 has recently emerged as an important player in neuronal dysfunction and degeneration and its activation has been linked to several neurodegenerative conditions such as AD, HD and stroke [16], [17], [18], [19], [20], [21], [22], [30], [37]. Direct inhibition of the enzyme as well as targeting of other players in the activation pathway might therefore be beneficial in these disorders. We show here that the cleavage of lamin A can be assessed in a high-throughput setting using a high-content imaging platform, making it an ideal intracellular readout to test interventions that decrease caspase-6 activity.

## Materials and Methods

All experiments were carried out in accordance with protocols (Animal protocol A07-0106) approved by the UBC Committee on Animal Care and the Canadian Council on Animal Care.

Ac-VEID-Afc, Ac-DEVD-Afc, Ac-VEID-CHO, zVAD-fmk and active caspase-3, -6 and -7 enzymes were purchased from Enzo Biosciences. Lamin A antibodies were from Cell Signaling Technology: cleaved lamin A and total lamin A/C for Western blotting (cat. nos. 2031 and 2032), cleaved lamin A for Mesoscale ELISA (cat. no. 2036) and immunofluorescence staining (cat. no. 2036). Pure lamin A protein and lamin B1 antibody were from Abcam (cat. nos. ab8982 and ab83472), antibody against full-length caspase-6 was from Cell Signaling Technology (cat. no. 9762), antibody against caspase-3 was from Cell Signaling Technology (cat. no. 9662), antibody against actin was from Chemicon (cat. no. MAB1501R). Camptothecin and staurosporine were from Sigma, cell culture reagents were from Gibco.

### Activity assays using Ac-VEID-AFC and Ac-DEVD-AFC

The amounts of enzyme indicated in the figures (5–500 nM) were incubated with 100 µM Ac-VEID-AFC at 37°C in caspase cleavage buffer (50 mM HEPES pH 7.4, 100 mM NaCl, 0.1% CHAPS, 1 mM EDTA, 10% glycerol, 10 mM DTT) for 1 h in a black 96well plate (Nunc). Fluorescence was measured every 5 min with excitation at 400 nm and emission at 505 nm. The initial linear part of the curve was analyzed.

For the calculation of active site concentrations, Ac-DEVD-AFC was used for caspases-3 and -7 according to the same protocol, and different amounts of zVAD-fmk were added to all reactions. The ratio between reaction velocity with inhibitor (Vi) over reaction velocity without inhibitor (V0) was plotted against the inhibitor concentration, and the concentration for y = 0 was determined as the active site concentration of the caspase.

The specific activity of the caspase enzymes used was determined with a standard curve using free AFC and the Ac-VEID-AFC or Ac-DEVD-AFC substrate for caspase-6 and caspases -3 and -7, respectively. The resulting specific activities were: 11.7 nmol Ac-VEID-AFC/min/µmol caspase-6, 13.7 nmol Ac-DEVD-AFC/min/µmol caspase-3 and 9.9 nmol Ac-DEVD-AFC/min/µmol caspase-7.

For the assessment of VEID cleavage in cell lysates, lysates corresponding to 100 µg protein were mixed with an equal volume 200 µM Ac-VEID-AFC in 2× caspase cleavage buffer. Fluorescence was measured as described above.

### Determination of Km and Kcat

Different concentrations of Ac-VEID-Afc substrate (1–150 µM) were digested with 10 nM caspase-6 in caspase cleavage buffer for 1 h in a black 96well plate (Nunc). Fluorescence was measured every 5 min with excitation at 400 nm and emission at 505 nm. The initial linear part of the curve was analyzed.

Different concentrations of lamin A protein (3–400 nM) were digested with 20 nM caspase-6 in caspase cleavage buffer for 15, 30, 45, and 60 minutes at 37°C. The reactions were stopped by shock-freezing, and thawed samples were analysed with the Mesoscale ELISA system. The cleavage rate was calculated at each lamin concentration and plotted against the concentration of Lamin A protein to calculate the Km and Kcat (Fig. S2).

### Caspase-6 activity assays using the Mesoscale ELISA system

100 ng pure lamin A protein was subjected to digestion by different concentrations of caspase enzymes as indicated in the figures (0.05–1000 nM) in caspase cleavage buffer for 30 min at 37°C. The reactions were stopped by shock-freezing, and thawed samples were analysed with the Mesoscale ELISA system: 5 µl of each reaction, corresponding to 25 ng lamin A protein, were spotted onto Mesoscale ELISA plates. Samples were incubated at room temperature for 1 h, then 150 µl 5% BSA in PBS was added per well and the plate was again incubated for 1 h at room temperature. All wells were briefly washed 3 times with 150 µl PBS containing 0.05% Tween-20, and 25 µl antibody mix was added per well (cleaved lamin A antibody, Cell Signaling 2036, 1∶100, goat anti-mouse sulfo-tag secondary antibody, MSD technology, 1∶500 in 1% BSA/PBS). After 1 h incubation at room temperature, all wells were briefly washed 3 times with 150 µl PBS containing 0.05% Tween-20, and 150 µl 2× reading reagent (MSD technology) was added per well. The plates were then read on a Mesoscale platform electrochemiluminescence reader (MSD technology) according to manufacturer's instructions.

For assays using MEF or MCF-7 cell lysates, lysates were diluted in PBS to 0.2 µg/µl, 5 µl were spotted in each well of a Mesoscale ELISA plate and the plate was developed as described above.

For assays using neuronal or transfected COS7 cell samples, lysates corresponding to 20 µg total protein were mixed with 100 ng lamin A protein in 1× caspase cleavage buffer. Samples were incubated for 3 h at 37°C, 5 µl were spotted in each well of a Mesoscale ELISA plate and the plate was developed as described above.

### Mouse embryonic fibroblast culture and lysis

Mouse embryonic fibroblasts (MEFs) from a C6ko mouse and its C6wt littermate were generated from day 12.5 embryos resulting from timed-pregnant heterozygous breedings and tissues not used for culture were genotyped. The generation and characterization of C6ko mice is described elsewhere [27]. Single pups were dissected in ice cold PBS, the body without head, limbs and liver, lung and heart was minced, the centrifuged pellet was digested with 0.25% Trypsin-EDTA at 37°C for 15 min and neutralized with MEF medium (Dulbecco's modified Eagle medium with high glucose, 10% fetal calf serum, 2 mM L-Glutamine, 100 µM non-essential amino acids, 1 mM sodium pyruvate, 1 µM β-mercaptoethanol). Cells were passaged through a pipette tip, centrifuged and suspended in MEF medium containing DNase I. After centrifugation, cells were suspended in fresh medium, incubated for 2 min at room temperature to let debris and cell clumps settle and cells in the supernatant were seeded into cell culture flasks. At passage 2, cells were immortalized by transfection with pSV3-neo SV40 large T antigen (ATCC) with the Fugene reagent (Roche) according to manufacturer's instructions. Immortalized cells were selected and propagated through the addition of 600 µg/ml G418 to the medium.

For the activation of caspase-6, cells were stressed with 50 nM staurosporine for 0–6 h and harvested by trypsinization. Cell pellets were lysed in 50 mM Tris pH 8, 150 mM NaCl and 1% Igepal with 4.2 mM Pefabloc and ‘Complete’ protease inhibitor cocktail (Roche) on ice, and protein concentrations were determined in the cleared lysates after centrifugation.

### Neuronal culture and lysis

Cortical neuronal cultures were prepared as described previously [38] from E16.5 littermate embryos obtained from timed-pregnant heterozygous breedings and tissues not used for culture were genotyped. Cultures were maintained at 37°C under 5% CO_2_ and half of the culture media was exchanged every 4–5 days. At day 10 *in vitro*, 5 µM camptothecin was added to the media to induce caspase activation [39], and cells were harvested after 30 h of treatment by scraping in ice-cold PBS supplemented with protease inhibitors (4.2 µM Pefabloc and ‘Complete’ protease inhibitor cocktail (Roche)). Cells were pelleted by centrifugation and stored at –80°C until lysis. Cell pellets were lysed in 50 mM HEPES pH 7.4, 100 mM NaCl, 1% Igepal, 1 mM EDTA and 10% glycerol with 4.2 µM Pefabloc and ‘Complete’ protease inhibitor cocktail (Roche) on ice, and protein concentrations were determined in the cleared lysates after centrifugation.

### Culture, transfection and lysis of COS7 cells

COS7 cells were cultured in Dulbecco's modified Eagle medium supplemented with 10% fetal calf serum and 2 mM L-Glutamine. Cells were transfected with human caspase-6 cDNA with a C-terminal DDK tag using the Fugene reagent (Roche) according to manufacturer's instructions. Cells were harvested 0–24 h after transfection by trypsinization. Cell pellets were lysed in 50 mM HEPES pH 7.4, 100 mM NaCl, 1% Igepal, 1 mM EDTA and 10% glycerol with 4.2 µM Pefabloc and ‘Complete’ protease inhibitor cocktail (Roche) on ice, and protein concentrations were determined in the cleared lysates after centrifugation.

### Culture and lysis of MCF-7 cells

MCF-7 cells were cultured in Dulbecco's modified Eagle medium supplemented with 10% fetal calf serum and 2 mM L-Glutamine. Cells were stressed with 5 uM camptothecin for different amounts of time and harvested by trypsinization. Cell pellets were lysed in 50 mM HEPES pH 7.4, 100 mM NaCl, 1% Igepal, 1 mM EDTA and 10% glycerol with 4.2 µM Pefabloc and ‘Complete’ protease inhibitor cocktail (Roche) on ice, and protein concentrations were determined in the cleared lysates after centrifugation.

### Western blotting

20 ng of pure lamin A protein was subjected to digestion by different concentrations of caspase-3, -6, and -7 (0.75–6 µM) in caspase cleavage buffer for 30 min at 37°C. The reactions were stopped by shock-freezing, and thawed samples were run on 4–12% Bis-Tris gels (Nupage, Invitrogen), transferred to PVDF membranes by electroblotting and membranes were developed with primary antibodies in 5% BSA/PBS. Fluorescently labelled secondary antibodies and the LiCor Odyssey Infrared Imaging system were used for detection.

For Western blotting of cell lysates, 50 µg total protein were run on 4–12% Bis-Tris gels (Nupage, Invitrogen) and processed as described above.

### Cell fixation and immunofluorescent staining

C6wt and C6ko MEFs were seeded at 2500 cells per well into 96well plates. N = 3 for each treatment. The following day, cells were treated with camptothecin (5 µM final) for 16 hours. Post stress, cells were washed in PBS, fixed in 4% formaldehyde (EMS)/PBS for 60 minutes at 4C, washed with PBS, and permeabilized with 0.3% Triton-X 100/PBS. Primary antibody to mouse cleaved lamin A (Cell Signaling Technology) was added at 1∶100, in normal goat serum, and incubated at 4C overnight. Plates were washed twice in PBS before adding secondary antibodies, AlexaFluor 488 anti-mouse IgG (Invitrogen), each at 1∶800 in normal goat serum, and DNA staining dye, Hoechst 33342 (Invitrogen), at 1∶10000, for 90 minutes at room temperature. Plates were washed again twice and left in PBS.

### Image capture and immunofluorescence data analysis

Labeled cells were analysed using the ThermoFisher Cellomics ArrayScan VTI, a high content scanning (vHCS) microscope, using version 6.6.2.0 software. The ArrayScan VTI captures images using an ORCA-ER camera in two channels applicable to the assay. XF-53 filters for 405 and 488 nm were used in automated image analysis to quantify nuclei number, and cleaved lamin A, respectively. The Cellomics toolbox, *Compartmental Analysis*, used an algorithm to encircle perinuclear staining of cleaved lamin A. The primary object in the Hoechst channel was identified as the nucleus of the cell of which 500 total were counted per well. In channels 2, the perinucleus was identified as a “circ”, which was 3 pixels outside the primary object, where the algorithm measured pixel intensity above background staining. The “MEAN_AvgCirIntensity” for each channel was used for analysis, under the vHCS analysis tool, and exported to Microsoft Excel spreadsheets and GraphPad Prism for further analysis.

## Supporting Information

Figure S1 **Active site titrations for caspases -3, -6, 7.** The exact active site concentration of each caspase used was determined by titrating the enzymes against the pan-caspase inhibitor zVAD-fmk [27].
(TIF)

Figure S2 **Km determination for lamin A.** **A:** The indicated concentrations of lamin A protein were digested with 20 nM caspase-6 and samples were analysed with the Mesoscale ELISA system. **B:** Cleavage rates were determined as the slope of the curves in (A) and plotted against the lamin A concentration to obtain values for Km and kcat through curve fitting using the built-in function of the GraphPad Prism 5.0 software package.
(TIF)
