# Supplementary figures and images for: A Quantitative Method for the Specific Assessment of Caspase-6 Activity in Cell Culture
Source: PLoS One. 2011 Nov 29;6(11):e27680. doi: 10.1371/journal.pone.0027680 (PMC3226564; doi:10.1371/journal.pone.0027680)

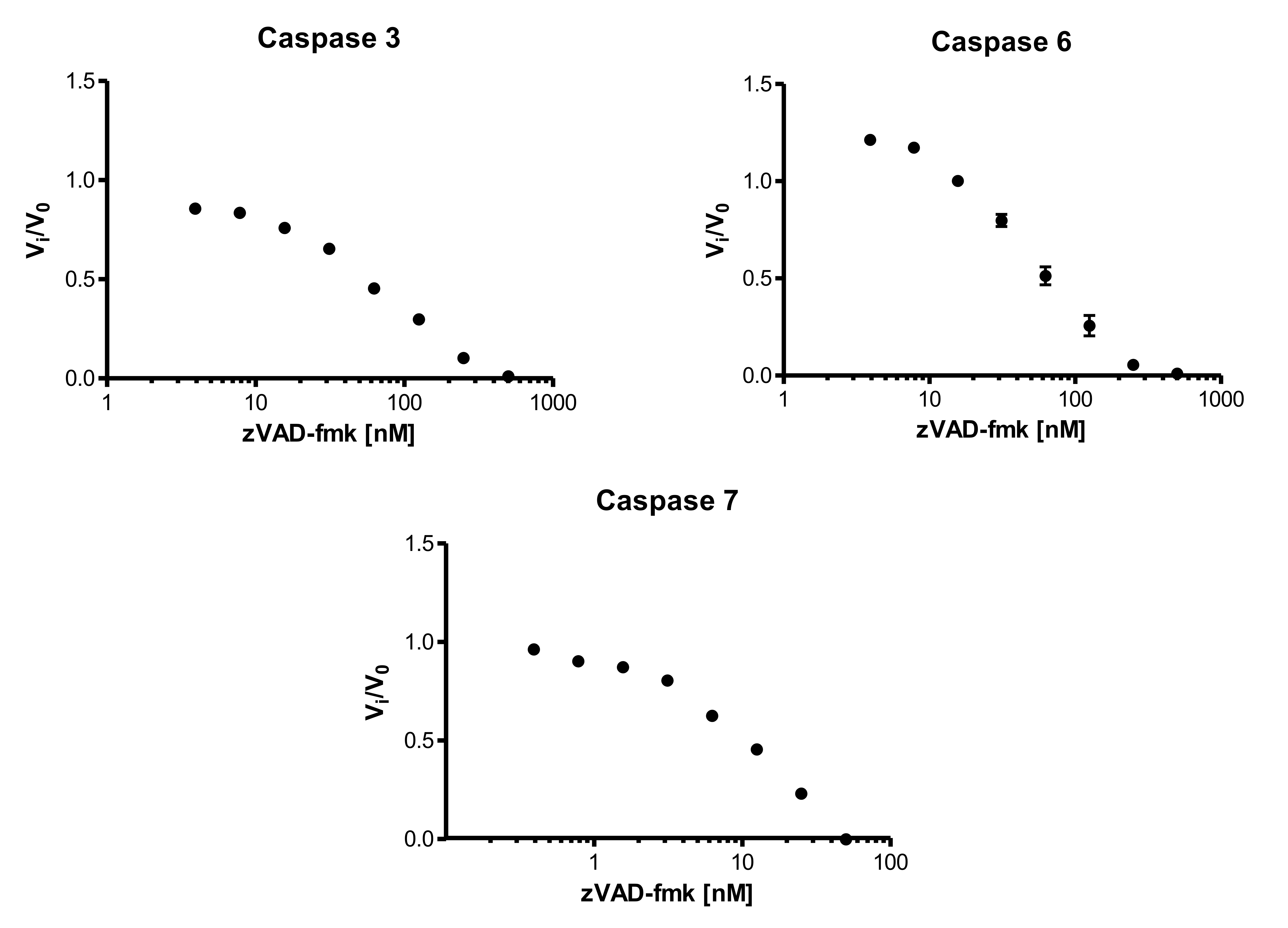

Supplement: Figure S1 — Active site titrations for caspases -3, -6, 7. The exact active site concentration of each caspase used was determined by titrating the enzymes against the pan-caspase inhibitor zVAD-fmk [27]. (TIF) [file pone.0027680.s001.tif]

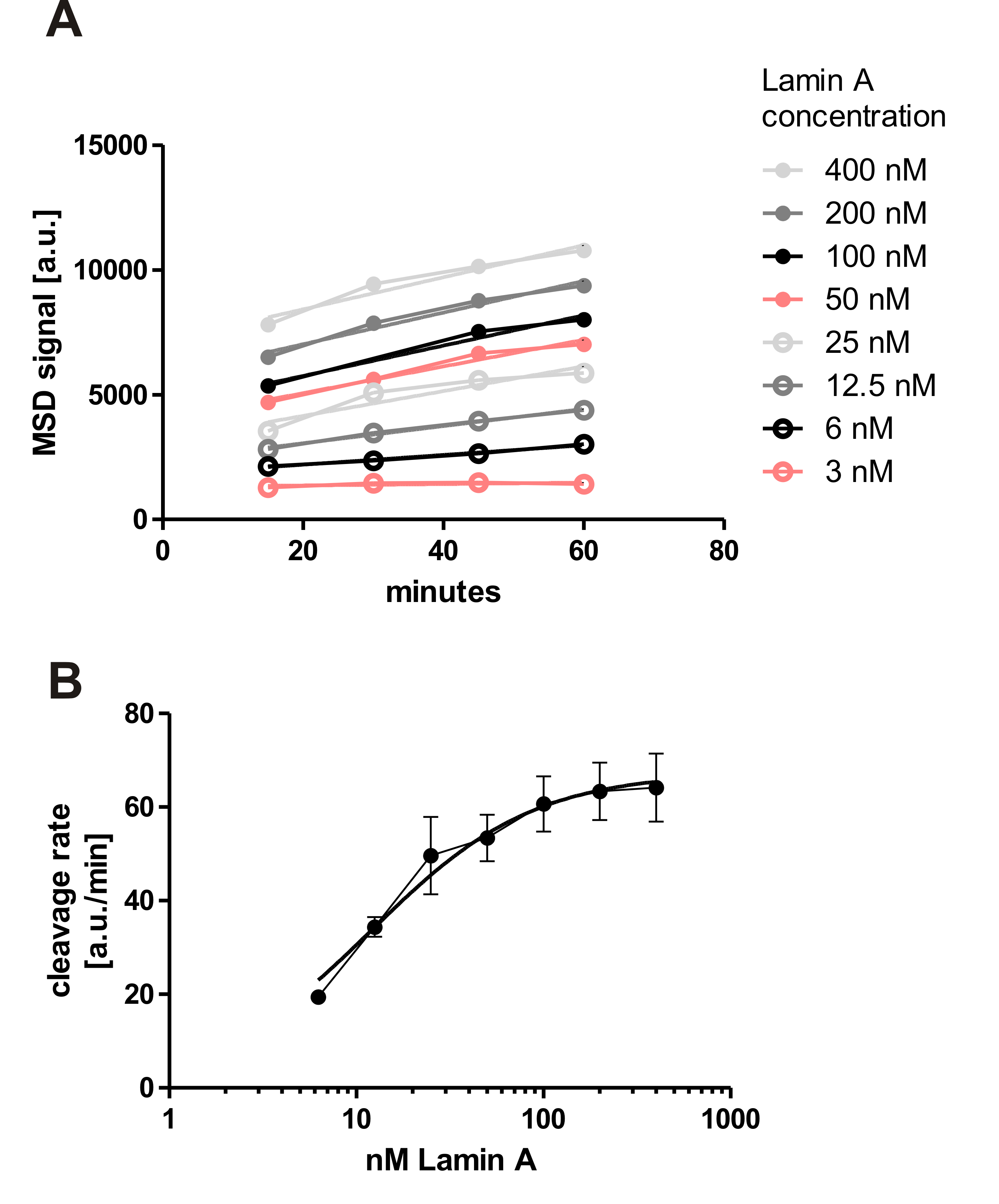

Supplement: Figure S2 — Km determination for lamin A. A: The indicated concentrations of lamin A protein were digested with 20 nM caspase-6 and samples were analysed with the Mesoscale ELISA system. B: Cleavage rates were determined as the slope of the curves in (A) and plotted against the lamin A concentration to obtain values for Km and kcat through curve fitting using the built-in function of the GraphPad Prism 5.0 software package. (TIF) [file pone.0027680.s002.tif]
